# Small bowel perforation with ingestion of a fish bone: case report

**DOI:** 10.11604/pamj.2024.48.94.42796

**Published:** 2024-07-09

**Authors:** Maissa Jallali, Hanen Zenati, Asma Korbi, Mohamed Ali Chaouch, Sadok Ben Jabra, Ibtissem Korbi, Faouzi Noomen

**Affiliations:** 1 Department of Visceral and Digestive Surgery, Monastir University Hospital, Monastir, Tunisia,; 2 Department of Gynecology, Monastir University Hospital, Monastir, Tunisia

**Keywords:** Foreign bodies, ingestion, intestinal perforation, case report

## Abstract

The perforation of the gastrointestinal tract caused by fish bone is rare, with a percentage rate of 1%. Surgical intervention is necessary in less than 1% of cases. We report a case of a 55-year-old male patient who was admitted for a rectus sheath abscess caused by perforation of the small bowel by a fish bone. He was treated surgically. Diagnosing perforation secondary to fish bone ingestion poses challenges due to its presentation.

## Introduction

Foreign body ingestion (FBI) is a relatively common problem, particularly among young children and older adults. The majority of ingested objects pass through the gastrointestinal tract (GIT) without causing any issues [1]. The risk of complication caused by FBI is very rare with a percentage rate of 1%. Complications can range from mild inflammation to life-threatening conditions like abscesses, perforation, obstruction and bleeding [2]. Even without serious complications, is an important aspect to consider because patients may experience anxiety, fear, or trauma related to the incident.

Fish bone (FB) perforation is one of the most common complications and can occur in all segments of the gastrointestinal tract, although it tends to happen more frequently in regions with acute angulation [2]. The diagnosis and management may have some difficulties [2]. We report a case of a 55-year-old male patient who was admitted for small bowel perforation due to ingestion of a foreign body and he was treated surgically. The postoperative course was simple and he was discharged after five days. Bowel perforation due to the ingestion of foreign bodies should be considered in the differential diagnosis of acute abdomen. Careful examination in front of this situation has a significant impact on the type of management.

## Patient and observation

**Patient information:** a 55-year-old male patient, with no medical or surgical history, presented to the emergency department with localized swelling in the left upper quadrant of the abdomen accompanied by pain. No local injection of any substance was reported by the patient.

**Clinical findings:** vital signs were normal and the patient was afebrile. Physical examination revealed a palpable mass in the left upper quadrant of the abdomen associated with localized inflammatory signs.

**Timeline of the current episode:** the patient's symptoms had been evolving for two days.

**Figure 1:**
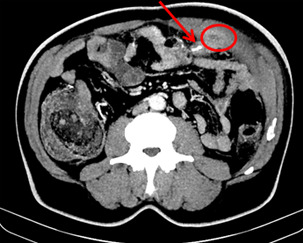
collection in the left rectus sheath (red circle) alongside a dense linear foreign body (red arrow) (axial slices)

**Figure 2:**
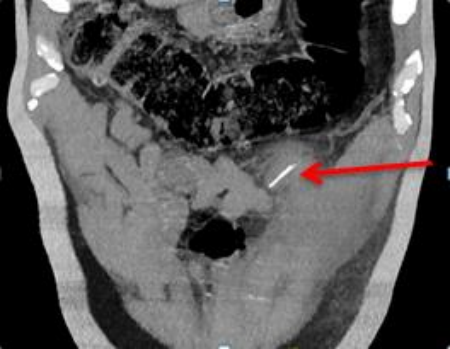
collection in the left rectus sheath alongside a dense linear foreign body (red arrow) (coronal slices)

**Figure 3:**
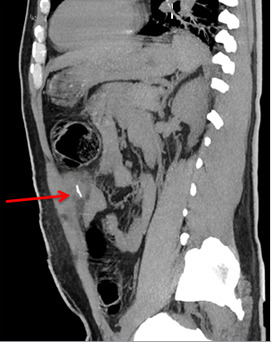
collection in the left rectus sheath alongside a dense linear foreign body (red arrow) (sagittal slices)

**Diagnostic assessment:** laboratory results showed an elevated white blood cell count of 15,750/uL, and an elevated C-reactive protein of 48 mg/dl A multi-detector Computed Tomography (MD-CT) showed a dense foreign body just below the abdominal wall measuring 23mm in continuity with the left abdominal wall associated with abscessed collection measuring 34-36mm. No peritoneal effusion was reported in the MD-CT (Figure 1, Figure 2, Figure 3).

**Diagnosis:** with this new information, we asked the patient if he had consumed any type of fish these past few days, and he recalled the accidental ingestion two days before he was admitted at the emergency department.

**Therapeutic interventions:** the patient underwent an emergent surgery and underwent a laparoscopy. Operative findings showed some adherences between the distal jejunum and the left abdominal wall. No perforation was found along the small bowel, and thus we suggested that the perforation was clogged by the abdominal wall. The abscess was located in the left rectus muscle and was successfully evacuated and the residual cavity was thoroughly washed. After the evacuation of the abscess, we found a linear foreign body corresponding to a fish bone. Passive drainage was placed inside the residual cavity and the post-operative course was uneventful.

**Follow-up and outcome of interventions:** the postoperative course was simple. The patient was discharged after five days.

**Patient perspective:** the patient was satisfied with symptom improvement and pleased with the surgery.

**Informed consent:** written informed consent was obtained from the patient for the publication of this case report and accompanying images.

## Discussion

Accidental ingestion of foreign bodies is a common condition in clinical practice and it can present with various clinical symptoms [1,2]. There are numerous causes for the ingestion of FB: the wearing of dentures, intellectual disability, prisoners, alcohol and drug abusers, suicide attempts, and carelessness among children [3]. Fish bones rank among the most ingested foreign objects by accident. Typically, their ingestion manifests with vague symptoms and the majority are expelled from the gastrointestinal system without any noticeable signs [3].

Complications occur in less than 1% of cases [4]. Although complications are rare, they can be serious and require medical intervention. These complications span from mild inflammatory changes to more severe outcomes such as abscess formation, perforation of the viscera, intestinal obstruction, and bleeding [5]. The severity of complications can vary depending on factors such as the size and shape of the bone, the location of the lodgment, and the individual's health status. Ignoring the potential risks associated with fish bone ingestion can lead to delayed diagnosis and treatment, which may exacerbate the situation. The perforation can occur in any segment of the digestive tract but is more frequent in areas of acute angulation such as the ileocecal area, the rectosigmoid junction and the esophagus [6].

Many symptoms can be presented following FB perforation and they are not specific such as generalized peritonitis, abscess formation, localized peritonitis, inflammatory mass, colorectal and colo-vesical fistulas, mechanical bowel obstruction and gastrointestinal hemorrhage [5]. As in our patient, he presented with symptoms not specific to bowel perforation which are abdominal pain and parietal abscess. There are several case reports of small bowel perforation caused by a fish bone, but with a rectus sheath abscess as the first clinical symptom, it is exceptional [5-7].

Most patients won't recall any accidental ingestion of a foreign body, especially amongst the elderly, and thus cross-examination may not be useful. Therefore, abdominal computed tomography (CT) is very useful for the diagnosis, especially for non-metallic foreign bodies. It can not only show the region of the perforation in most cases for example: thickened intestinal wall or localized pneumoperitoneum, but can also identify the calcified FB [8]. For fish bone material, the typical image is of a rectilinear calcified image surrounded by inflammation. Retrospective study in Singapore reviewed 22 cases of FB perforation and 15 of them were caused by a fish bone. Out of these 15 cases, seven of them underwent a preoperative abdominal CT scan. All of these seven cases showed a linear calcified lesion with inflammation or abscess formation. Although the CT scan is essential for the diagnosis, its limitations are dominated by lack of the observer awareness and the scanning thickness. In this case, the diagnosis and management may have some difficulties [8].

The approach to managing bowel perforation is not standardized and it can involve either surgical or nonsurgical interventions. Surgery and endoscopic therapy are the most commonly used treatment modalities. Surgery is not mandatory in clinically stable patients with small and contained perforations. FBs located in the jejunum or ileum are risk factors for both complications and surgery. Indications for surgical management encompass situations such as peritonitis resulting from perforation, the presence of abscesses, severe inflammation, or significant bleeding [6]. Non-surgical management of bowel perforation depends on the size and the location of perforation, diagnosis time, patient condition and contamination degree. It involves implementing strategies such as nutrition support, intravenous fluid administration, broad-spectrum antibiotics, controlling the source of contamination and endoscopic therapy [9]. The duration of antibiotic therapy varies, with some physicians opting for a 7-14-days course, while others base it on white blood cell (WBC) levels or the clinical presentation. Presently, a common practice is to administer antibiotics for 5-7 days if there is clinical improvement in 82 patients [10].

Our case demonstrates the rare presentation of a rectus sheath abscess secondary to an accidental ingestion of a fish bone in a 55-year-old male with no medical history, treated by surgery. We advise a thorough and careful examination in front of this situation, as it has a significant impact on the type of management.

## Conclusion

Diagnosing perforation secondary to fish bone ingestion poses challenges due to its presentation resembling other medical conditions and the uncertainty surrounding the time of ingestion. However, advancements in medical imaging have significantly enhanced sensitivity and specificity in detecting fish bones. The management of complications can be either medical or surgical, contingent upon factors such as the presence of complications, their location and the overall clinical picture of the patient. This case underscores the need to include foreign body perforation in the differential diagnosis of acute abdomen, especially for seemingly straightforward presentations.
